# The Catastrophic HPV/HIV Dual Viral Oncogenomics in Concert with Dysregulated Alternative Splicing in Cervical Cancer

**DOI:** 10.3390/ijms221810115

**Published:** 2021-09-18

**Authors:** Rahaba Marima, Rodney Hull, Georgios Lolas, Konstantinos N. Syrigos, Minah Kgoebane-Maseko, Andreas Martin Kaufmann, Zodwa Dlamini

**Affiliations:** 1 SAMRC Precision Oncology Research Unit (PORU), Pan African Cancer Research Institute (PACRI), University of Pretoria, Pretoria 0028, South Africa; rodney.hull@up.ac.za (R.H.); glolas@med.uoa.gr (G.L.); mw.kgoebane-maseko@up.ac.za (M.K.-M.); andreas.kaufmann@charite.de (A.M.K.); 2 3rd Department of Medicine, National and Kapodistrian University of Athens, 15772 Athens, Greece; ksyrigos@med.uoa.gr; 3 Clinic for Gynecology, Charité-Universitätsmedizin Berlin, Corporate Member of Freie Universität Berlin and Humboldt-Universität zu Berlin, Berlin Institute of Health, Charité Campus Virchow Klinikum, Augustenburger Platz 1, 13353 Berlin, Germany

**Keywords:** human papillomavirus (HPV), human immunodeficiency virus (HIV), alternative splicing, cervical cancer, oncovirus, highly active antiretroviral therapy (HAART)

## Abstract

Cervical cancer is a public health problem and has devastating effects in low-to-middle-income countries (LTMICs) such as the sub-Saharan African (SSA) countries. Infection by the human papillomavirus (HPV) is the main cause of cervical cancer. HIV positive women have higher HPV prevalence and cervical cancer incidence than their HIV negative counterparts do. Concurrent HPV/HIV infection is catastrophic, particularly to African women due to the high prevalence of HIV infections. Although various studies show a relationship between HPV, HIV and cervical cancer, there is still a gap in the knowledge concerning the precise nature of this tripartite association. Firstly, most studies show the relationship between HPV and cervical cancer at genomic and epigenetic levels, while the transcriptomic landscape of this relationship remains to be elucidated. Even though many studies have shown HPV/HIV dual viral pathogenesis, the dual molecular oncoviral effects on the development of cervical cancer remains largely uncertain. Furthermore, the effect of highly active antiretroviral therapy (HAART) on the cellular splicing machinery is unclear. Emerging evidence indicates the vital role played by host splicing events in both HPV and HIV infection in the development and progression to cervical cancer. Therefore, decoding the transcriptome landscape of this tripartite relationship holds promising therapeutic potential. This review will focus on the link between cellular splicing machinery, HPV, HIV infection and the aberrant alternative splicing events that take place in HIV/HPV-associated cervical cancer. Finally, we will investigate how these aberrant splicing events can be targeted for the development of new therapeutic strategies against HPV/HIV-associated cervical cancer.

## 1. Introduction

Histologically, cervical cancer can be divided into two main types, squamous cell carcinoma (SCC) and adenocarcinoma (AC). SCC is the most common type accounting for over 70% of all cases [1,2]. Cervical cancer can be attributed to various risk factors, and these include smoking, alcohol consumption, multiple sexual partners, unprotected sex, socioeconomic status, a family history of cervical cancer, extended use of oral contraceptives, human immunodeficiency virus (HIV) infection and persistent human papillomavirus (HPV) infection [3,4,5,6]. However, persistent HPV infection is the key contributor to the development of cervical cancer [1,7]. HPV infections can be classified as either low-risk or high-risk, with over 200 genotypes documented [8]. HPV 16 and 18 are the most dominant high-risk genotypes responsible for the development of invasive cervical cancer [9,10,11]. Persistent high-risk HPV infection is responsible for over 90% of cervical cancers while prophylactic vaccination against HPV infections including high-risk genotypes can reduce cervical cancers [10].

In sub-Saharan Africa (SSA), HIV positive women have increased HPV prevalence and cervical cancer incidence compared to HIV negative women. This may be partly attributed to HIV’s altering effect on HPV pathogenesis. Furthermore, HIV positive women have increased risk of HPV infection [12]. Globally, and in SSA, cervical cancer is the leading cause of mortality. SSA has elevated the dual burden of both HPV and HIV infection [13,14]. HIV infection is linked to higher HPV infection rates, reduced HPV clearance, more severe cervical precancerous lesions and higher risk of progression to invasive cervical cancer [15,16]. HIV positive women have been reported to have almost two times higher cervical cancer mortality compared to HIV negative women [17,18]. The advent of highly active antiretroviral therapy (HAART) has improved the life expectancy of HIV positive people, and HIV positive women. As an AIDS defining cancer (ADC), there is still a growing need for cervical cancer prevention, particularly in those countries with a high HIV/AIDS infection rate as well as in low-to-middle-income countries (LTMICs) [19]. In the USA, cervical cancer incidence and prevalence have been reduced by highly effective cervical cancer screening programs and HPV vaccination. A comprehensive background knowledge of the molecular mechanisms involved in the dual HPV/HIV infection promoting cervical cancer pathogenesis can aid in the development of informed cervical cancer management, policy and decision-making. This is especially true for HIV positive women. To date, various studies have evaluated the HPV/HIV interplay pathogenesis, while the gap in the understanding of this dual viral molecular pathogenesis is still extensive. While HAART reduces the incidence of other ADCs, the relationship between HAART and cervical cancer is still poorly understood [20].

Several extrinsic dynamics pose a threat in the fight against cervical cancer in LTMICs. Such factors include gender inequality, inequitable laws and inappropriate traditional practices and violence between intimate partners. These factors are unfavourable to women, limit women’s access to information, education, employment, opportunities and access to social and health services. Increased HPV and HIV co-infection has been frequently documented. Unfortunately, African women are mostly affected by this catastrophic dual viral infection. Globally, young women <35 years in the sub-Saharan African (SSA) region make up ~60% of HIV infection prevalence and almost 70% of newly acquired infections in Africa. With no proper interventions in place, the World Health Organization (WHO) estimates that cervical cancer deaths will double to over 400,000 by 2030, and the SSA region will make up 90% of the cases resulting in mortality [17,19].

**Figure 1:**
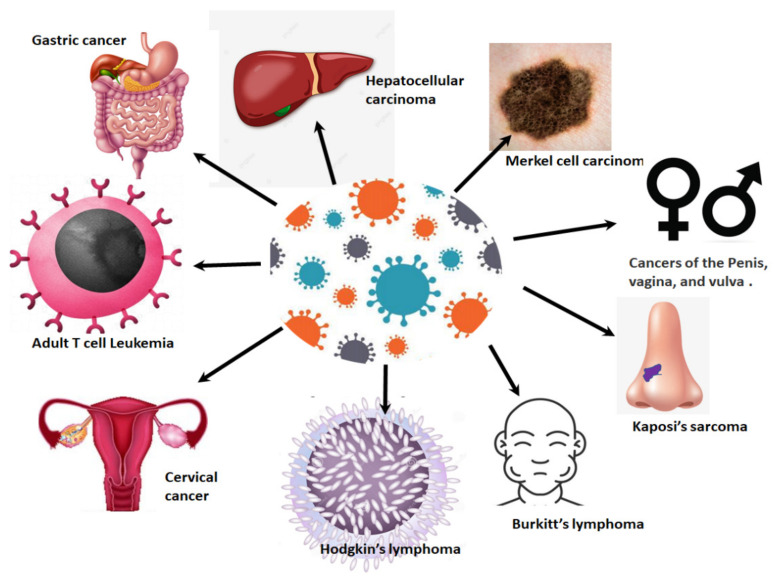
Viral-associated cancers: infections with viruses such as Epstein–Barr virus, HPV, hepatitis B virus, hepatitis C virus, human herpes virus, human T cell leukemia virus and HIV can lead to the development of a variety of cancers.

Cancers associated with viral infections are on the rise globally, and in SSA [18,21] (Figure 1). Approximately a third of all cancers in Africa are linked to a viral etiology [22]. Viruses such as HPV are generally dependent on the cellular machinery of the host for replication [18]. As a result of this, the splicing machinery of the host is also compromised upon HPV infection. As an oncovirus, HPV infection will result in the synthesis of splice variants of viral and cellular mRNA that favour cell survival mechanisms such as excessive cell proliferation, immune response evasion and inhibition of tumour suppressor proteins [3,23]. This review focuses on the link between cellular splicing machinery, HPV/HIV infection and the aberrant alternative splicing events that take place in HIV/HPV-associated cervical cancer. Finally, we investigate how these aberrant splicing events can be targeted for the development of new therapeutic strategies against HPV/HIV-associated cervical cancer.

## 2. Cervical Cancer Epidemiology

**Figure 2:**
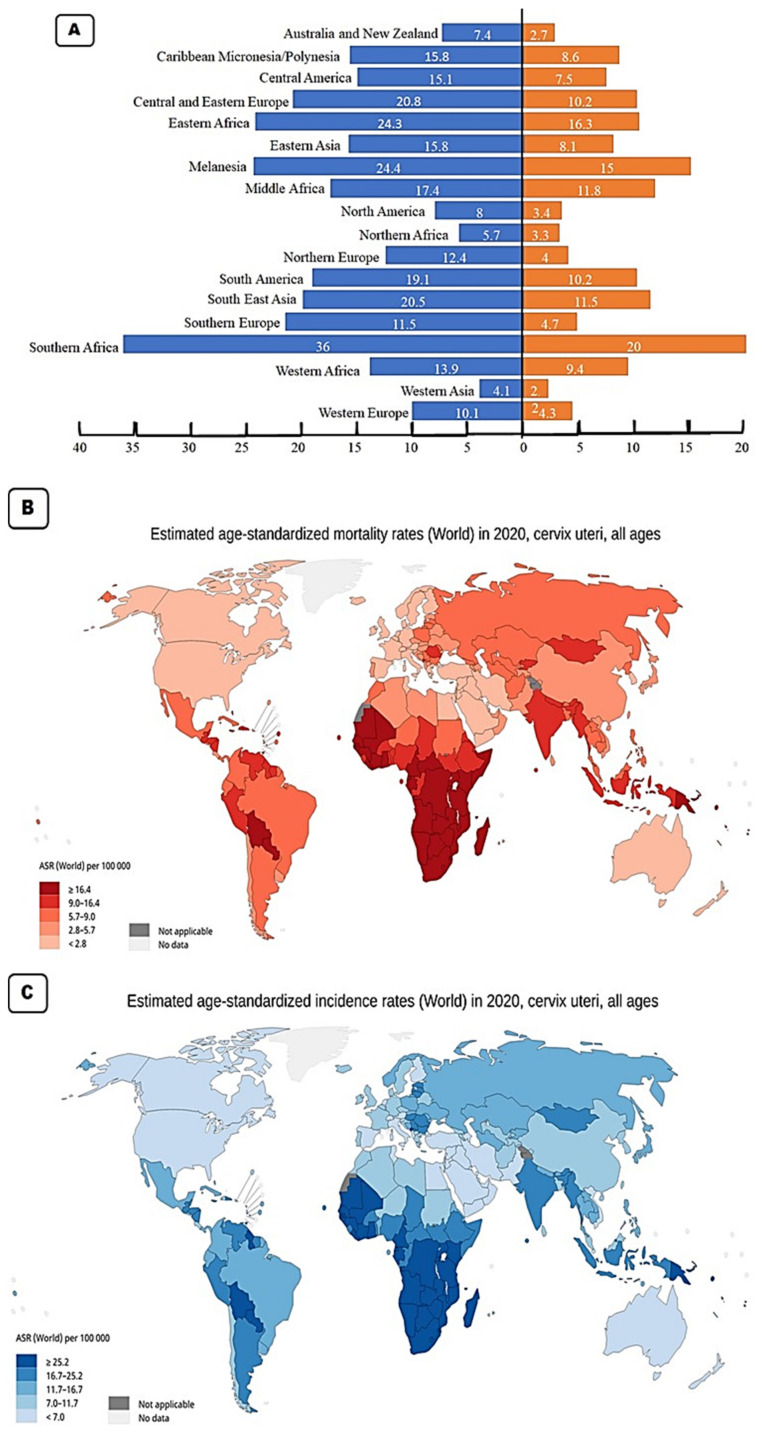
(**A**) Age standardised rates (ASR) (per 100,000 women per year) incidence and mortality for cervical cancer in specific regions. (**B**) Map showing the age standardised rate (ASR) for incidence in individual countries. (**C**) Map showing the age standardised rate (ASR) for mortality in individual countries [24].

With a worldwide age standardised incidence rate (ASR) of 15.6 per 100,000 per year, cervical cancer is the fourth most diagnosed cancer in women worldwide. The three cancers most common in women are breast, colorectal, and lung cancer. Based on the 2020 GLOBOCAN stats, the Southern African region has the highest incidence rates with an ASR of 36 per 100,000 (Figure 2A). This region also has the highest mortality rate of 20 per 100,000 as a result of cervical cancer (Figure 2B). All the countries in this region have incidence rates above 35 cases per 100,000 (Figure 2C). The country with the highest incidence rate is Eswatini with an ASR for incidence rates of 84.5, followed by Malawi (ASR 67.9), Zambia (ASR 65.5), Zimbabwe (ASR 61.7), Lesotho (56.8), Mozambique (ASR 50.2), Angola (ASR 37.5), Namibia (ASR 37.4), South Africa (ASR 35.3) and the country in the region with the lowest incidence, Botswana with an ASR of 34.4 (Figure 2B). The geographical regions with the second highest incidence rate for cervical cancer are Melanesia and the Eastern African region. Melanesia includes countries such as Fiji, Vanuatu, the Solomon Islands, and Papua New Guinea. The Eastern African region includes countries such as Burundi, Kenya, Rwanda, Tanzania, and Uganda. These two regions have an ASR of 24.3–24.4. Eastern Africa has the higher mortality rate with an ASR of 16.3 compared to that of Melanesia (ASR 15) (Figure 2A,B). The area with the third highest incidence rate for cervical cancer is Central and Eastern Europe (ASR 20.8), followed closely by Southeast Asia (ASR 20.4). The country with the highest incidence rate in Eastern and Central region is Romania (ASR 22.6) while Indonesia has the highest incidence rate in Southeast Asia with an ASR of 24.4 [24].

The region with the next highest incidence rate is South America with an ASR of 19.1 per 100,000, followed by Middle Africa (ASR 17.4), Eastern Asia and the Caribbean (both with an ASR of 15.8), Central America (ASR 15.1), Western Africa (ASR 13.9), Northern Europe (ASR 12.1), Southern Europe (ASR 11.5) and Western Europe (ASR 10.1). The Western Europe region contains countries such as the UK with an incidence rate of 9.9 and a mortality rate of 1.9 (Figure 2C). It also includes France with an incidence rate of 7 and a mortality rate of 2.2. These low incidence and low mortality rates are typical in developed nations. The regions with the lowest incidence of cervical cancer include North America (ASR 8), Australia and New Zealand (ASR 7.4), North Africa (ASR 5.7) and the region with the lowest incidence rate is West Asia with an incidence rate of 4.1 and a mortality rate of 2 per 100,000. In the North American region, the USA has an incidence rate of 6.2 per 100,000 and a mortality rate of 2.1 per 100,000 [24].

## 3. HPV Pathogenesis

More than 200 genotypes of HPV based on their sequence can be divided into α, β, γ, δ, µ genera [4,6]. The HPV E1 and E2 proteins are early viral proteins required for the viral DNA replication and protein synthesis. In addition, E2 also regulates the expression of E6 and E7, while E4 and E5 help in viral assembly and stimulation of cellular proliferation. The late proteins, L1 and L2 form the minor and major capsid proteins [25].

HPV infects the squamous epithelial cells, which have the ability to proliferate and gain access to basal cells during abrasion or trauma. HPV then induces the viral genes’ expression, which is critical for viral replication in the basal cells. The HPV interaction with the host cells occurs via heparin sulfate proteoglycans and host cell surface receptors such as α-6 integrins. The E2 protein acts as a transcriptional repressor of E6 and E7. HPV replicates by the rolling circle mechanism, during which it may integrate into the human genome. This integration perturbs the expression of E2, thereby favouring the upregulation of the expression of the E6 and E7 oncoproteins, leading ultimately to cell transformation. Following viral replication, L1 and L2 proteins form the virus capsid and the subsequent mature virus production. The mature virus is then released with the help of the E4 protein and desquamation of cells [5,6,7].

## 4. HPV-Related Cancers

**Figure 3:**
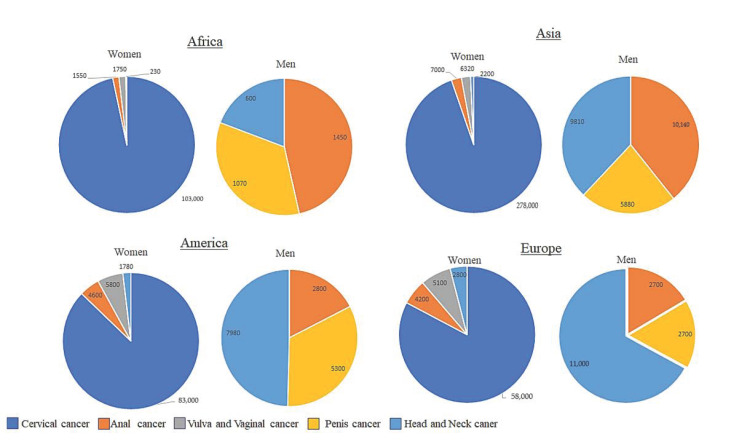
HPV-related cancers in Africa, Asia, America and Europe. Regardless of the continent, cervical cancer is the most common HPV-associated cancer in women. Amongst men, Africa is different from America, Asia and Europe as anal cancer is the most common HPV-related cancer in Africa. In Asia, head and neck cancer and anal cancer are the most common HPV-related cancers. In America and Europe, the most common HPV-related cancer is head and neck cancer.

Cancers caused by HPV arise in tissues and organs where HPV infects epithelial cells. These tissues contain squamous cells that line the inside of these organs and are present within these tissues. It is these cells that are the target of HPV infection. The sites of HPV infection include the oropharynx, anus, penis, vagina, vulva and most importantly for this review, the cervix (Figure 3). With some geographical variation, HPV infection causes 70% of cancers of the vulva, 75% of all vaginal cancers, 60% of all penile cancers, 90% of all anal cancers and 70% of all oropharyngeal cancers. In terms of cervical cancer, it is thought that nearly all cervical cancers are caused by persistent HPV infection [3,26].

The association between HPV, HAART components and cervical cancer in HIV positive women remains poorly understood. It has been reported that initiating HAART early and attaining sustained adherence is more likely to reduce SIL (squamous intraepithelial lesion) and cervical intraepithelial neoplasia (CIN), and thus reduce the incidence of invasive cervical cancer [15]. Furthermore, the long-term effects of HAART on cervical cancer progression in HIV positive women is also unknown. Kelly et al. (2017) reported that HIV positive women on HAART showed reduced prevalence of invasive cervical cancer [27]. This study is in concordance with Clifford et al. (2016) [15]. However, conflicting reports stated that increasing incidence of invasive cervical cancer occurred in patients receiving HAART, probably because longer survival provides the time window for cervical cancer to develop [28]. The lack of adequate studies from African populations in particular, poses a challenge in attempting to decipher the effects of HAART on invasive cervical cancer. There have been a few African studies indicating that there is an inverse relationship pattern between invasive cervical cancer incidence and the length of time the patient has been undergoing HAART [20,27,29,30]. Conversely, the few studies performed in Latin American and Asian studies reveal a paradoxical relationship between HAART use and incidence of invasive cervical cancer [31,32,33].

HPV has been classified as a direct carcinogen while HIV has been classified as an indirect carcinogen through immunosuppression. The carcinogenic classification of these viruses was done by the International Agency for Research on Cancer [1,34].

## 5. Alternative Splicing

### 5.1. Alternative Splicing and Its Implications in Cervical Cancer

Alternative splicing (AS) is an important physiological process that contributes to proteome diversity. The protein isoforms formed from various mRNA transcripts of the same gene may have similar or opposing functions. The AS process is tightly regulated by various small nuclear ribonucleoproteins (snRNP) and heterogeneous nuclear ribonucleoproteins (hnRNPs). The two protein families are trans-regulatory to the AS process and their regulation is crucial in cellular homeostasis. In various cancers, including cervical cancer, these regulatory elements have been reported to be altered [35,36,37]. Upregulated snRNPs favour alternative splicing, while overexpression of hnRNPs inhibits AS. Evidence of HPV-mediated AS in cervical cancer has been documented [3].

### 5.2. HPV and Splicing

**Figure 4:**
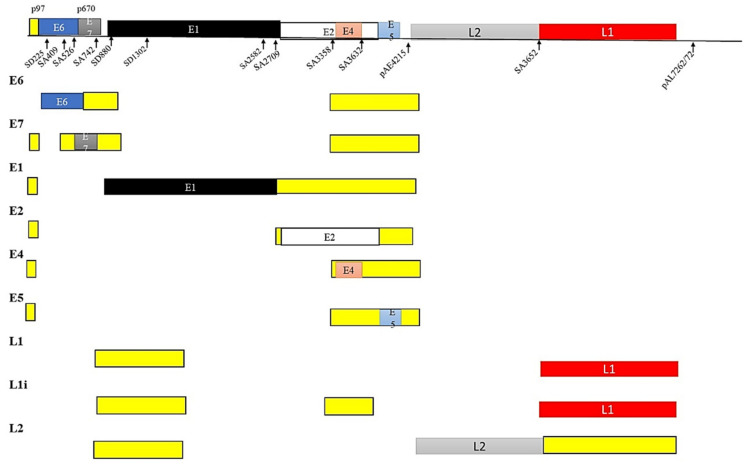
Splicing of the HPV genome. The DNA genome of HPV is made up of the long control region (LCR, not shown), the early region (E) and the late region (L). The LCR regulates transcription and replication. The genes of the early region control infection and virus expression, while genes of the late region code for the virus capsule proteins. Alternative splicing in HPV infection results from two different promoters in low-risk HPV and by a single promoter in high-risk HPV. Different isoforms of the E6 protein are found in high-risk HPV.

The HPV genome is a circular double stranded (ds) DNA genome of about 8 kb and has three regions, namely, (a) the long control region (LCR), (b) the early region and (c) the late region. The LCR is responsible for the regulation of transcription and replication, the early region is responsible for viral infection and establishing the virus in the host cell, while the late region is responsible for viral encapsulation by coding for the L1 and L2 proteins [34,38]. E6 and E7 splice variants are usually correlated with high-risk HPV-associated cancers [39] (Figure 4).

The LCR of HPV is the regulatory region that controls transcription of the virus. The early promoter responsible for early gene expression is located on the E6 ORF, while the E7 ORF is responsible for late gene expression. Differential transcription strategies are employed by E6 and E7 in high-risk and low-risk HPVs, indicating the significance of alternative splicing in HPV infection. In low-risk HPV, the E6 and E7 genes are transcribed by two different promoters, while in high-risk HPV E6 and E7 are transcribed by a single promoter from a polycistronic pre-mRNA [36]. In high-risk HPV 16, E6 alternative splicing results in different isoforms, E6*I, E6*II, E6*III, E6*IV, E6*V, E6*VI, E6^E7, E6^E7*I and E6^E7*II. Additionally, E6*I, E6*II, E6*III, E6^E7 are different E6 isoforms identified in HPV 18 [39,40,41]. Amongst these isoforms, E6*I and E6*II are high-risk transcripts often upregulated in cervical cancer and associated with advanced disease [31,33,39,42,43,44].

### 5.3. HPV and Alternate mRNA Splicing

The E2 and E6 proteins of HPV 16 interrupt RNA splicing using multiple ways. Firstly, through the preferential binding of E2 and E6 C-terminus to the pre-mRNA introns. Secondly, these two proteins (E2 and E6) bind to splicing factors such as SRSF4, 5, 6, 9. It is also interesting to note that this interaction between E2 and E6 and these SR proteins occurs at the same site as RNA binding [45,46]. SR proteins negatively regulate alternative splicing by intron-exon recognition. The binding of E2 and E6 to SR proteins disrupts the splice site recognition. This interruption may lead to abnormal splicing events in the host. Furthermore, HPV E4 protein binds to serine-arginine protein kinases (SRPK) 1 that phosphorylate SR proteins. This E4/SRPK1 interaction results in the inhibition of SR protein activation [37,46,47].

The aberrant activity of both SR proteins and hnRNPs in cervical cancer has been demonstrated to elicit the production of oncoproteins by processing pre-mRNA transcripts that are either derived from the human genome or from the HPV genome. Both SR proteins and hnRNPs have been shown to control the production of viral oncoprotein isoforms that are key in the HPV life cycle and cell transformation. Protein isoforms generated through alternative splicing are expressed selectively in a time-dependent and tissue-specific manner. This allows for the regulation of various metabolic pathways involved in cell proliferation, differentiation, cell cycle control, and cell death [48,49].

### 5.4. HIV and Alternate mRNA Splicing

HIV also regulates the host splicing machinery, thereby advancing the progression of the viral infection. The altered splicing leads to the expression of viral genes responsible for invasion, replication and assembly of the virus. Contrary to HPV, HIV-related malignancies evolve as a result of immunosuppression that may lead to co-infection by other oncogenic viruses such as HPV. As a retrovirus, HIV depends completely on the cellular machinery of the host for amplification.

**Figure 5:**
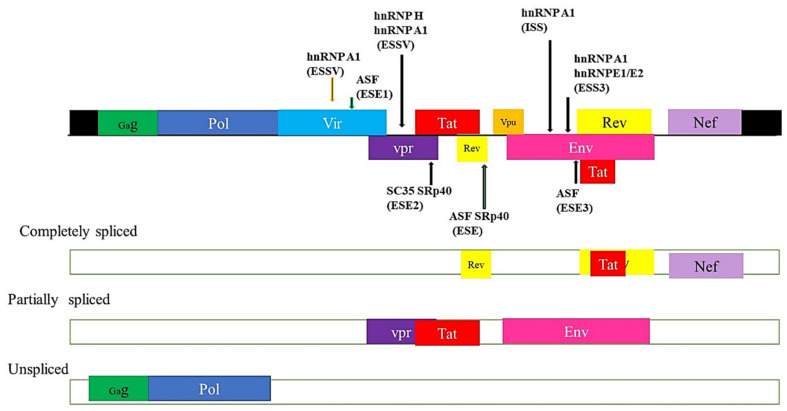
Splicing of the HIV genome. In the early stage of HIV infection, tat, rev and nef regulatory proteins are transcribed from completely spliced mRNA. Additionally, following early infection, vif, vpr, vpu and env are transcribed from partially spliced mRNA. Furthermore, non-spliced mRNA is translated to form the gal and pol structural proteins.

The HIV genome codes for 10 proteins with various functions, all of which are important for the HIV lifecycle, Figure 5. HIV produces various mRNA transcripts of the pre-mRNA through alternative splicing. These mRNA variants (gag, pol, vif, vpr, vpu, env, nef, rev and tat) are important for the expression of the HIV protein isoforms. These HIV distinct isoforms may exist in full form, intron-lacking variants and intron-comprising variants [50,51,52,53]. Strict alternative splicing of these isoforms is crucial for the HIV life cycle and infection. HIV pathogenesis is severely affected when the RNA export pathway is interrupted. Based on their sizes, the HIV-1 mRNAs are classified into full-length 9 kb, intron containing 4 kb and intron-less 2 kb mRNA classes. In the early stage of HIV infection, tat, rev and nef regulatory proteins are transcribed from a completely spliced mRNA. Additionally, following early infection, vif, vpr, vpu and env are transcribed from a partially spliced mRNA. Furthermore, non-spliced mRNA is translated to form the gal and pol structural proteins [54]. HIV exploits the host splicing machinery to maintain its potency. Decoding alternative splicing events associated with HIV and HPV infection in cervical cancer may be a promising source for the development of future therapeutic interventions. The 4 kb intron containing gag and pol is mainly spliced from the major splice donor (D1) to one of the acceptors within the central cluster, while the 2 kb intron-less mRNA transcript is spliced from the central splice donor (D4) to the terminal acceptor (A7). Finally, the minor, additional 1 kb mRNA transcript variant was discovered through next generation sequencing (NGS) [53,55].

### 5.5. RNA Splicing Factors

The splicing process comprises of sequential reactions that involve the recruitment of spliceosome components and interaction with the cis-acting regulatory intronic sequences such as the 50 splice donor (SD) site and the 30 splice acceptor (SA) site, the intervening branch point and the polypyrimidine tract [56]. The alternate recognition of various splice donor and acceptor sites permits the production of differential mRNA isoforms. The pre-mRNA maturation is regulated by the trans-acting splicing factors, which include hnRNPs. Both constitutive and alternative splicing deregulation play critical roles in carcinogenesis [48,49,57,58].

### 5.6. Splicing Factors Deregulation in Cervical Cancer

Both SRSFs and hnRNPs have been reported to be upregulated in the basal and middle layers of the cervical epithelium, while being downregulated in the terminal differentiated layers [37]. For example, hnRNP A1 is upregulated in high-grade CIN or in cervical cancer compared to normal epithelium of the cervix. In SiHa cervical cancer cells, hnRNP A1 was reported to facilitate the production of mature miR-18a, promoting proliferation and invasion of these cells. Furthermore, miR-18a inhibits the expression of PTEN, WNK2, BTG3, SOX6 and RBSP3 genes by binding to their 3′ UTR transcripts [35,39]. Additionally, siRNA knockdown of hnRNP A2/B1 was shown to inhibit cell proliferation, cell migration and invasion, while upregulating apoptosis in HeLa and CaSki cervical cancer cells [59].

In addition, high levels of hnRNP H and I have been reported in cervical cancer and high-grade CIN compared to normal tissues. Knockdown of hnRNP I was shown to reduce the proliferation of HeLa cells. Furthermore, the lncRNA ARAP1-AS1 binds with PTB-associated splicing factor (PSF) and causes the release of hnRNP I, which in turn, enhances c-Myc expression and subsequent cell proliferation while inhibiting apoptosis of CaSki and SiHa cervical cancer cells [53,60].

Both SRSF9 and SRSF 10 have also been shown to be overexpressed in cervical cancer compared to normal epithelium. Interestingly, E6/E7 silencing in CaSKi cervical cancer cells results in the downregulation of SRSF 10 [11,25,45,58].

## 6. HPV, Genome Instability and DNA Damage Response (DDR)

**Figure 6:**
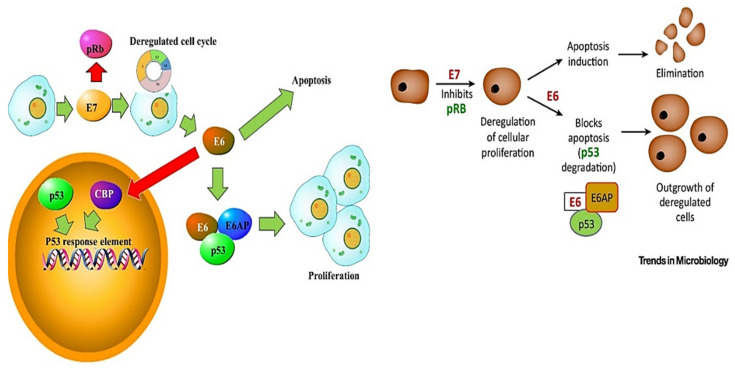
DDR and apoptosis signalling involving E6 and E7. E6 targets p53 by forming a ternary complex with E6-associated protein (E6AP), inducing the degradation of p53. Additionally, the E6 protein binds the transcriptional co-activator cAMP response element binding protein (CREB) binding protein/p300 (CBP/p300), downregulating the ability of CBP/p300 to activate p53 responsive elements. Furthermore, E7 prevents cell cycle inhibition. E7 also binds and inhibits the retinoblastoma (pRB) protein family, interfering with the pRB/E2F interaction. Normally, E2F induces the transcription of S phase genes such as cyclins A and E.

Many oncogenic viruses such as HPV may affect the DNA damage response pathway (DDR) and cell cycle regulation. Different studies have previously addressed the effect of specific HPV proteins on DNA repair pathways. This begins with the dependency of HPV on the host machinery to amplify its genome. To achieve this, HPV must induce the host to re-enter the DNA replication S-phase of the cell cycle [61]. This is achieved by the action of the E6 and E7 oncoproteins on the cell cycle regulating factors. These viral oncoproteins, (Figure 6) work in synergy to dysregulate the cell cycle, immortalise primary cells and can also individually evade the mitotic checkpoint (MCC) and cell cycle arrest mediated by p53 in response to DNA damage [62,63,64].

E6 targets the p53 tumour suppressor protein. E6 alters p53 functional capacity by forming a ternary complex with E6-associated protein (E6AP), inducing its degradation by the ubiquitin-mediated proteolysis pathway [34,37,38]. Additionally, the HPV 16 E6 protein binds the transcriptional co-activator cAMP response element binding protein (CREB) binding protein/p300 (CBP/p300). This results in E6 downregulating the ability of CBP/p300 to activate p53 responsive elements in the promoters of p53-regulated genes [33]. Furthermore, the E6 oncoprotein delays primary cells’ senescence by the upregulation of the human telomerase reverse transcriptase (hTERT). In addition, E6 promotes HPV oncogenesis by dysregulating other cellular processes such as apoptosis, cell differentiation and cell polarity [62]. In a similar manner, the E7 protein from high-risk HPV binds to and deregulates the retinoblastoma (pRB) protein family. This E7/pRB interaction interferes with the pRB/E2F interaction. Normally, E2F induces the transcription of S phase genes such as cyclins A and E [6,44]. By binding to E2F, E7 downregulates the expression of growth arrest factors such as the cyclin dependent kinase inhibitors (CDKIs) p21 and p27, thereby driving the progression of the cell cycle. The E7 protein from high-risk HPV has also been shown to interact with histone deacetylases 1 and 2 (HDAC1 and HDAC2), altering the gene expression pattern of affected cells. Furthermore, HPV oncoproteins have been reported to target the ataxia telangiectasia mutated (ATM) and ataxia telangiectasia and Rad3-related (ATR) DNA damage response (DDR) pathways. For example, HPV 18 E7 has been reported to induce elevated levels of phosphorylated ATM and downstream kinases such as checkpoint kinase 1 (CHK1) [65]. Additionally, DNA topoisomerase 2-binding protein 1 (TopBP1) knockdown suppresses ATR, thereby suppressing HPV31 replication. TopBP1 acts upstream of ATR. ATR and ATM proteins are constitutively activated in HPV positive keratinocytes. Blocking the ATR/CHK1 pathway in these keratinocytes is linked to the downregulation of HPV replication and downregulates the expression of late genes. HPV infection may upregulate the frequency of DNA alterations and reduce their removal in the host cell by targeting the host’s DDR. Furthermore, the HPV-associated impairment of the cell cycle checkpoints and apoptosis by HPV oncoproteins favour cell survival mechanisms. Ultimately, these persistent effects on the host cells may lead to cellular alterations, giving rise to precursor lesions, and possibly leading to malignant progression. While errors in the DDR pathways may contribute to genomic instability and possibly lead to tumour progression, such DDR alterations also hold promising therapeutic opportunities [66]. In particular, the involvement of post-transcriptional gene regulation such as alternative splicing is emerging as a potent therapeutic target [67]. Mechanistic studies showing the molecular mechanisms of HPV oncoproteins dysregulation of the DDR pathways’ alternatively spliced mRNA transcripts in cervical cancer are further required to better comprehend HPV-cervical cancer oncogenesis.

## 7. Conclusions

The SSA region has the highest burden of dual HPV/HIV viral infections, exacerbating the cervical cancer pandemic in this region. Poor diagnostic and prognostic tools in LTMIC are obstacles in the efforts to manage cervical cancer. Although preventative measures are common in developed countries, the high burden of the already infected women with both HPV and HIV urgently warrants interventions [1]. There is a growing need to combat cervical cancer carcinogenesis. Therefore, the development of therapeutic strategies aimed to target alternative splicing, particularly for the already infected women is of paramount importance to improve patient survival. In this respect, although it has been reported that HIV has a modifying effect on HPV pathogenesis, further studies to understand the HPV/HIV molecular relationship are required. Furthermore, while it has been reported that HAART decreases the incidence of other AIDS-related cancers, HAART’s relationship with cervical cancer is obscure [11,23].

HPV prophylactic vaccines have been identified as the most potent intervention method against cervical cancer. The licensed HPV vaccines currently showing safety, high efficacy and immunogenicity are 2-valent CervarixTM, 4-valent Gardasil and 9-valent Gardasil [68,69]. Recurrence or metastasis of cervical cancer is associated with poor prognosis and an overall 5-year survival rate of 17% [70]. In light of this, novel prognostic and therapeutic targets are urgently needed to combat the devastating effects of cervical cancer, particularly in the SSA region. Most molecular cervical cancer studies focus on alterations at the transcriptomic and epigenetic levels, with very few studies focusing on post-transcriptional gene regulation. It has been demonstrated that AS events in tumours are upregulated by 30% compared to healthy samples [71]. These results further corroborate the importance of alternate mRNA splicing as an exploitable candidate in cervical cancer therapy.
